# Analysis of host- and strain-dependent cell death responses during infectious salmon anemia virus infection *in vitro*

**DOI:** 10.1186/1743-422X-6-91

**Published:** 2009-07-01

**Authors:** Berit L Schiøtz, Espen S Bækkevold, Lene C Poulsen, Siri Mjaaland, Tor Gjøen

**Affiliations:** 1 Department of Pharmaceutical Biosciences, School of Pharmacy, University of Oslo, Norway; 2 Institute of Pathology, Rikshospitalet-Radiumhospitalet Medical Center, University of Oslo, Oslo, Norway; 3 Department of Food Safety and Infection Biology, Norwegian School of Veterinary Science, Oslo, Norway

## Abstract

**Background:**

Infectious salmon anemia virus (ISAV) is an aquatic orthomyxovirus and the causative agent of infectious salmon anemia (ISA), a disease of great importance in the Atlantic salmon farming industry. *In vitro*, ISAV infection causes cytophatic effect (CPE) in cell lines from Atlantic salmon, leading to rounding and finally detachment of the cells from the substratum. In this study, we investigated the mode of cell death during *in vitro *ISAV infection in different Atlantic salmon cell lines, using four ISAV strains causing different mortality *in vivo*.

**Results:**

The results show that caspase 3/7 activity increased during the course of infection in ASK and SHK-1 cells, infected cells showed increased surface expression of phosphatidylserine and increased PI uptake, compared to mock infected cells; and morphological alterations of the mitochondria were observed. Expression analysis of immune relevant genes revealed no correlation between in vivo mortality and expression, but good correlation in expression of interferon genes.

**Conclusion:**

Results from this study indicate that there is both strain and cell type dependent differences in the virus-host interaction during ISAV infection. This is important to bear in mind when extrapolating *in vitro *findings to the *in vivo *situation.

## Background

Infectious salmon anemia virus is an aquatic orthomyxovirus of the genus *Isavirus *[1]. ISAV is the causative agent of infectious salmon anemia (ISA), an emerging disease causing high mortalities and great economic losses in the Atlantic salmon (*Salmo salar *L.) farming industry. Large differences in disease severity and clinical signs are observed both in field outbreaks [2-5] and experimental trials [6-11]. Affected fish are often anemic; other typical findings are haemorrhagic liver necrosis, ascites and petechiae in the viscera [12]. The virus is reported to cause acute or protracted disease in fish *in vivo *[6]. Several strains of ISAV are known and categorized according to the highly polymorphic region of the hemagglutinin-esterase protein, and according to the ability of the virus to induce acute versus protracted disease in affected fish. Although much is known about the structure and genetics of the virus, less is known about the immune reactions induced by ISAV. *In vitro*, ISAV replicates and causes cytophatic effect. We wanted to investigate the mode of cell death and transcriptional changes after *in vitro *infection. We also wanted to compare stress responses induced by four different Norwegian strains of ISAV. It has previously been shown that the mechanism of cell death during ISAV infection is dependent on the cell type; that apoptosis is induced in CHSE-214 and SHK-1 cells, and that necrosis is the outcome after *in vitro *infection in TO cells, infected with Canadian isolates [13].

Apoptosis is described as an ordered process of cellular demise and can play a role in the innate cellular responses to limit virus infection [14]. Influenza virus is reported to induce apoptosis *in vivo *and *in vitro *[15-18]. Upon activation of the apoptosis machinery, cells undergo distinct morphological and biochemical changes, which include DNA fragmentation and condensation [19], blebbing of the plasma membrane and exposure of phosphatidylserine on the cell surface, marking the cells for phagocytosis [20], thus no inflammatory response is elicited. However, viruses have evolved mechanisms to manipulate the apoptotic machinery [21,22].

Apoptosis is an evolutionary conserved process, and many genes encoding homologues to annotated apoptosis proteins have been identified in fish [23,24]. Caspases, a family of cysteine proteases, are central in the process. They consist of initiator caspases (caspase -8 and -9), that relay death signals to effector caspases (caspase -3, -6 and -7). Effector caspases cleave a range of proteins involved in cell structure and function. Effector caspases have been identified in Atlantic salmon [25].

The mitochondria are central in the control of cell death, and numerous molecules involved in apoptosis are released from the mitochondrial intermembrane space and promotes apoptosis as a consequence of mitochondrial outer membrane permeabilization (MOMP). Mitochondria are often filamentous and arranged in a network, so called mitochondrial reticulum, in the cytoplasm. Being dynamic organelles, mitochondria can undergo fission and fusion, resulting in morphological changes [26]. Mitochondrial fission has been implicated in apoptosis [27].

There are several studies on RNA viruses showing an inverse correlation between degree of apoptosis *in vitro *with pathogenicity *in vivo *[28-30]. The molecular mechanisms leading to cell death upon ISAV infection is currently not known. Recently, we compared the gene expression pattern in cells after infection with two highly virulent ISAV strains [31]. We have now included two additional low virulent isolates and compare the degree of apoptosis and gene regulation induced by these four strains of ISAV showing different pathogenicity *in vivo *[6]. A range of morphological and biochemical assays were undertaken in an attempt to elucidate the molecular mechanism of ISAV induced cell death. We also investigated stress responses in two other cell lines from Atlantic salmon, SHK-1 and TO cells. Staurosporine (SS), a protein kinase inhibitor was used as a positive control for apoptosis induction [32,33]. In this study, we report both cell- and strain-dependent effects of ISAV on stress responses in cells from Atlantic salmon.

## Results

### Cell morphology and viral growth

**Figure 1:**
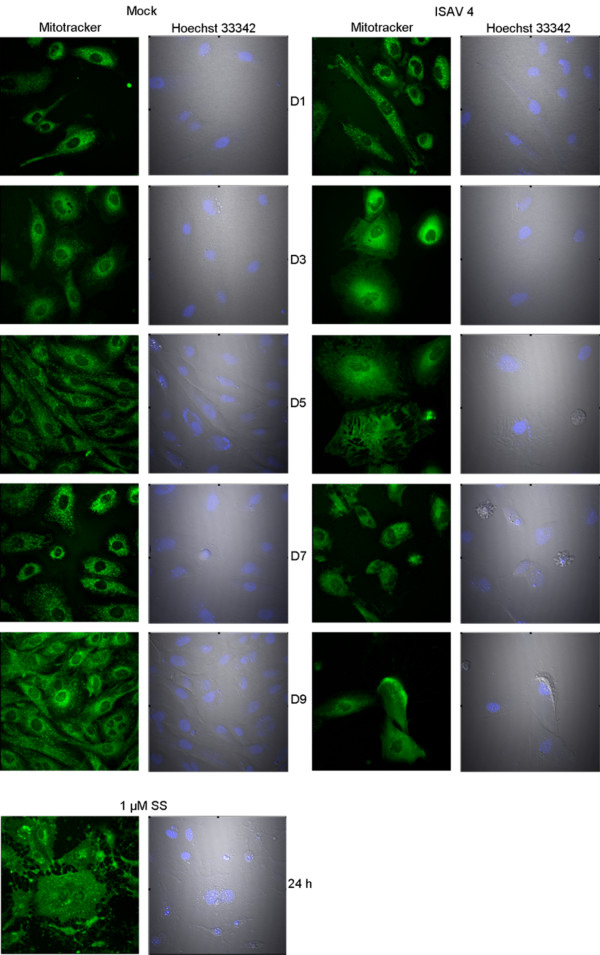
**Fragmentation of mitochondria in ISAV infected cells**. Morphology of mitochondria in ASK cells; control, infected with ISAV 4 or treated with 1 μM SS (lower panel). Cells were stained with Mitotracker and Hoechst 33342. Mitochondria of mock infected cells show an elongated, thread like morphology, while SS treated and ISAV infected cells, from day 3 p.i, show mitochondrial fragmentation. (40× magnification).

When ASK cells were infected at MOI of 1, cytopathic effect (CPE), characterized by cell shrinking, rounding and detachment from the substratum, was evident from day 5. The detachment of cells increased during the course of infection, as observed in ASK cells by DIC microscopy (figure 1, right panel). The end-point of the study was day 9, when few of the ISAV infected cells were left in the wells. To assay mitochondrial morphology, ISAV infected cells were stained with mitotracker. From day 3 post infection, the infected cells displayed grain-like mitochondrial morphology, compared to a more thread-like morphology of the control (figure 1). This was also true for cells treated with 1 μM staurosporine (SS) for 24 h. The staining pattern was similar in both live and fixed cells. When viral titers in supernatant were quantitated at day 5 and end of experiment we found no significant differences between viral strains (not shown).

### Cell viability

**Figure 2:**
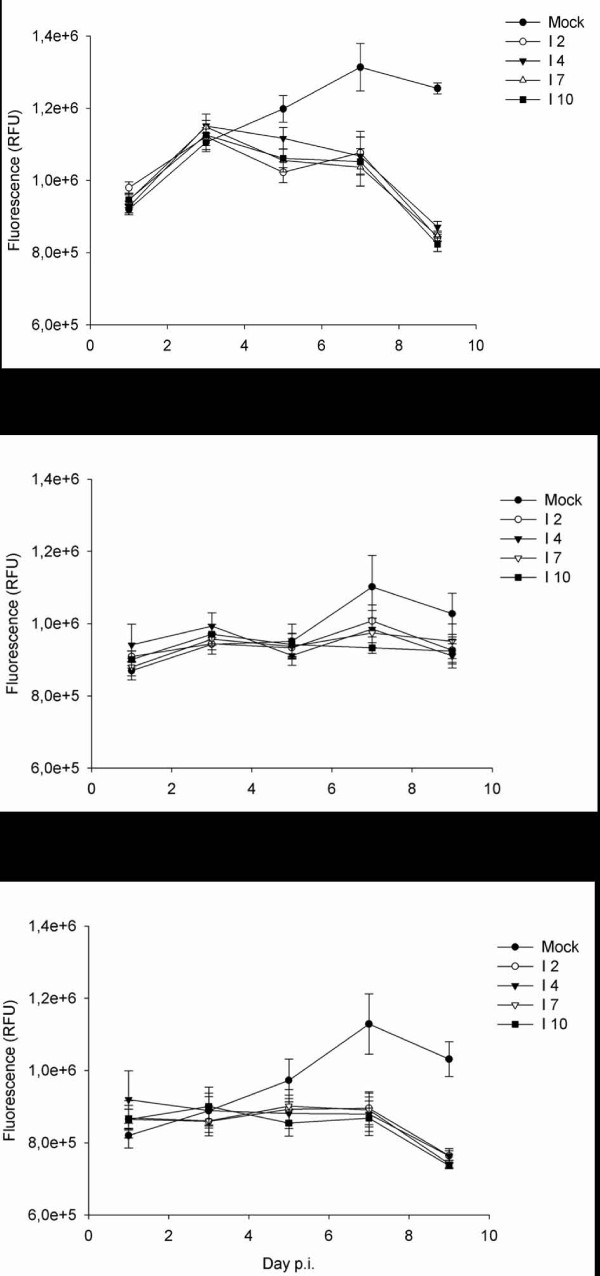
**Cell viability after ISAV infection**. Cell viability analyzed with the Cell Titer Blue assay. Relative fluorescence units (RFU) of SHK-1 (A), TO (B), and ASK (C) in control or ISAV infected cells, infected with ISAV 2, 4, 7 and 10, on days 1, 3, 5, 7 and 9 p.i.

Cell viability was assayed in SHK-1, TO and ASK cells infected with ISAV 2, 4, 7 or 10 during the course of infection (days 1, 3, 5, 7 and 9) using the Cell Titer Blue assay. The results, shown in figure 2A–C, show that after 7 days of infection, there was a reduction in viable cells compared to mock. The reduction in the number of non-infected cells at the end of the experiment is probably due to high cell density. The viability of mock-infected cells was statistically significant from cells infected with the 4 virus isolates. With the exception of day 1 vs.9, and day 3 vs. 5, the viability was significantly different from day to day.

### Caspase 3/7 activity

**Figure 3:**
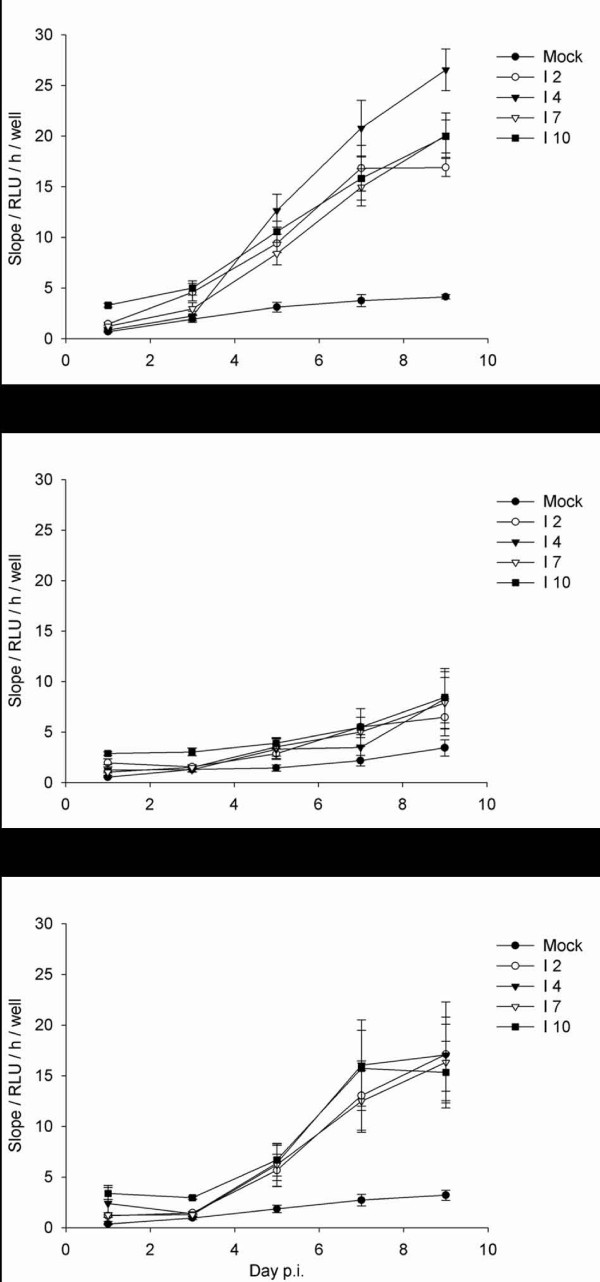
**Caspase activity after ISAV infection**. Caspase 3/7 activity, expressed as slope of relative luminescence units (RLU)/hour (h)/well in control or ISAV infected (ISAV 2, 4, 7 and 10) SHK-1 (A), TO (B), and ASK (C) cells, on days 1, 3, 5, 7 and 9 p.i.

It is well established that induction of the main effector caspase, caspase 3, is induced upon apoptosis in cells [34] The presence of inducible caspase activity was verified by incubation of the cells with SS. This treatment increased caspase activity up to 100 fold (data not shown). To assay if an ISAV infection with a MOI of 1 would induce effector caspase activity, and whether there were cell type or strain specific differences, Caspase 3/7 activity was measured in SHK-1, TO and ASK cells infected with ISAV 2, 4, 7 or 10 during the course of infection (days 1, 3, 5, 7 and 9). When the three different cell types were infected with ISAV at an MOI of 1, there was a time and cell dependent increase in caspase activity (figure 3A–C). This increase was significantly higher in SHK than ASK cells, which was higher than TO. There were no significant effects of viral strain on caspase activity (figure 3).

### DNA laddering

**Figure 4:**
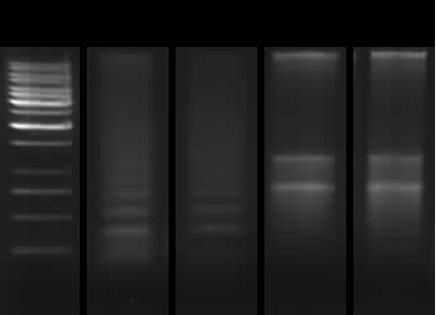
**DNA laddering after ISAV infection**. Agarose gel electrophoresis of DNA from ASK cells. Lane 1, 1 kb ladder; lane 2, positive control; lane 3, cells treated with 1 μM SS 24 h; lane 4, mock infected cells day 5 p.i.; lane 5; ISAV infected cells day 5 p.i. Laddering pattern was demonstrated by SS treated cells, in addition to positive control.

As 200 bp fragmentation of DNA is considered a hallmark of apoptosis, DNA was isolated and run on a gel for detection of the characteristic laddering pattern. Infection in neither of the three cell types with ISAV 4 on days 3, 5 or 7 led to DNA laddering, while laddering occurred in cells treated with SS. Gel electrophoresis of SS, mock and day 5 p.i. in ASK cells is shown in figure 4.

### TUNEL

**Figure 5:**
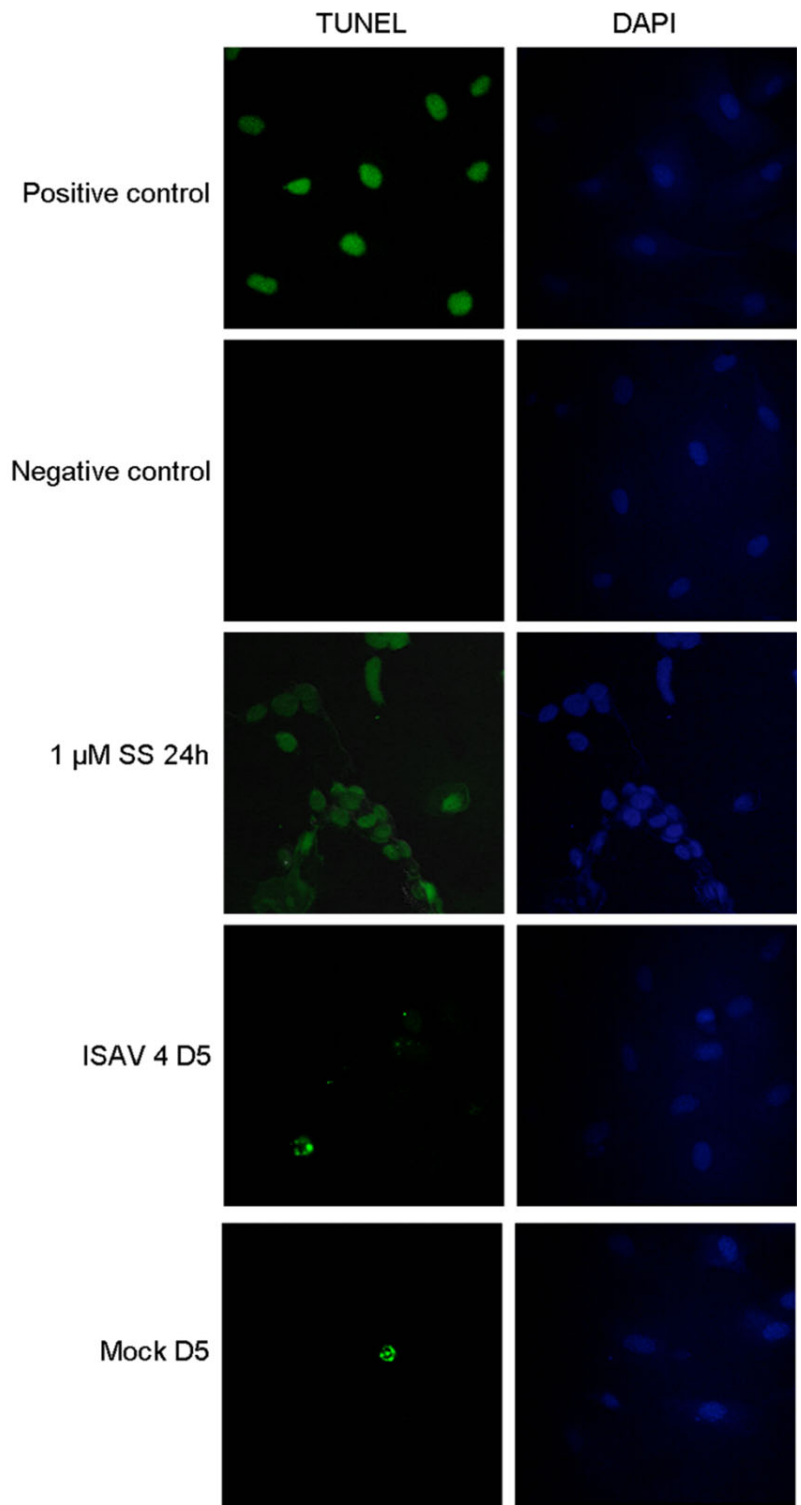
**TUNEL staining of ISAV infected cells**. TUNEL staining of ASK cells; positive control, negative control, staurosporine treated (1 μM SS 24 h), ISAV infected day 5 p.i. and mock infected day 5 p.i. (40× magnification).

Staining with TUNEL showed that all cells in the positive control (cells treated with DNAse) were positive, as well as cells treated with SS were positive for TUNEL. In mock and ISAV infected cells 3 or 5 days p.i. there were very few TUNEL positive cells (~1%), as shown in figure 5.

### Phospholipid distribution

**Figure 6:**
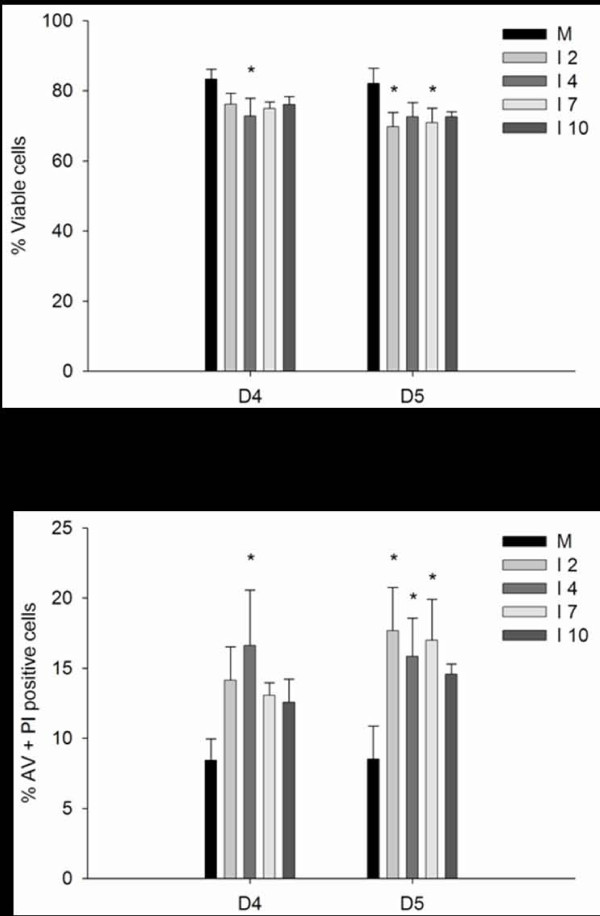
**Exposure of phosphatidsylserine in ISAV infected cells**. A. Representative AV/PI flow cytometric dot-plots of ASK cells. Left panel = mock, right panel = 5 days p.i. with ISAV 4. One representative experiment out of three is shown. The lower left quadrant represents viable cells (negative for both AV and PI). Early apoptotic ASK cells in the lower right quadrant exclude PI, demonstrating cytoplasmic membrane integrity. AV+PI positive cells are shown in the upper right quadrant considered to be late apototic or early necrotic cells. The upper left quadrant displays cells that take up PI, considered to be necrotic or dead cells. B. Percentages of cells in the quadrants regarded as viable (upper panel) and AV+PI positive (lower panel). Virus isolates statistically significantly different from mock is marked with an asterisk.

During apoptosis, membrane asymmetry can change, as phospholipids flip from the inside to the outside of the cell membrane. To assay whether these changes could be induced by *in v*itro ISAV infection in ASK cells, cells were stained with fluorescently labeled Annexin-V (AV) and co-stained with propidium iodide (PI). The cells were analyzed by flow cytometry and separated into four populations; viable cells, Annexin V positive cells, AV and PI positive cells and PI positive cells. A representative scatter of mock and ISAV infected cells is shown in figure 6a. The distribution of cells in each quadrant on days 4 and 5 p.i. from 3 experiments were subjected to two-way ANOVA analysis. Figure 6b shows the percentages in the quadrants with viable and AV+ PI positive cells ± SE, on days 4 and 5 p.i. ISAV infected cells showed a different distribution compared to the mock infected cells. A large proportion of the cells (80%) were characterized as viable. On day 5 p.i. there was a significant increase of double positive staining in infected cells (except for ISAV 10). There was no significant effect of viral strain. We also assayed PI uptake in the cells by fluorescence microscopy and found that adherent cells in the flasks, both infected and mock, did not take up PI. During flow cytometry, all nonadherent cells (mock and infected) were PI positive, whereas adherent cells, detached by trypsination did not take up PI (data not shown).

### Plasma membrane integrity

**Figure 7:**
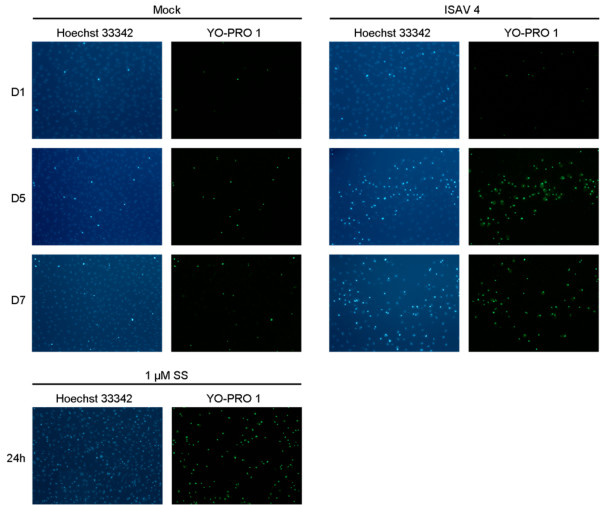
**Cell membrane integrity after ISAV infection**. Microscopic analysis of apoptosis with YO-PRO-1 staining. Cells were mock or ISAV infected, or treated with SS (1 μM 24 h) and stained with YO-PRO-1, Hoechst 33342 and PI, and examined under a fluorescence microscope at the indicated time points (10× magnification).

While an intact plasma membrane is characteristic of apoptotic cells, disruption and leakage is characteristic for necrotic cells [35]. Membrane permeability was therefore also assayed in this study. YO-PRO-1 dye is a nucleic acid stain that can enter apoptotic cells [36]. During the course of infection, the amount of YO-PRO-1 positive cells in ASK cells increased. Very few cells were PI positive; however there was a slight increase in PI positive cells during the course of infection (figure 7).

### Real-time PCR

**Figure 8:**
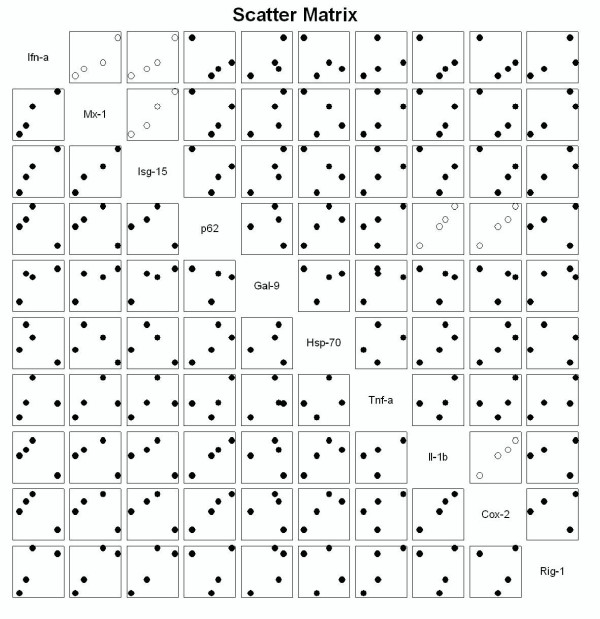
**Correlation between genes upregulated in QPCR after ISAV infection**. Scatter matrix plot of correlation between the 10 most upregulated genes in the QPCR analysis of ASK cells infected with four ISAV strains. In each plot, X-data corresponds to the second row, and Y-data corresponds to the first row in the analysis. Plots with open symbols indicate positive correlation (p > 0.05).

QPCR analysis of various genes previously demonstrated to be induced by ISAV [31,37] and other infectious agents in salmon [38-40], showed that all genes investigated were induced by at least one isolate (except for *Transaldolase-1 *which was down-regulated by all isolates). The genes that were differentially induced by viral strains were *Inf-α, Mx-1, Galectin-9, Ciap, Hsp-70 *and *Ogf-1*. Heat shock protein 70, was the only gene showing higher expression in cells infected with the low virulent strains ISAV 7 and 10, compared to cells infected with ISAV 2 and 4. We then tested if there were any correlation between in vivo mortality induced by the various strains [6] and in vitro gene expression (Pearson product moment correlation). There was no correlation between *in vivo *mortality and *in vitro *gene expression induced by the four strains. However, there was a strong positive correlation between the 3 interferon related genes *Ifn-α, Mx-1 and Isg-15 *expression in the 4 different groups of infected cells. A strong positive correlation was also found between the inflammatory markers *p62, Il-1β *and *Cox-2 *as shown in the scatter matrix in figure 8.

## Discussion

Virally induced cell death is an important aspect of viral pathogenesis. The ways viruses manipulate cell death mechanisms induced in the host are numerous and complex [21,22,41]. Influenza virus is shown to induce apoptosis both in cell culture and *in vivo *[15-18]. Also, several fish viruses induce apoptosis in host cells [42-44]. In this report we have employed multiple morphological and biochemical methods to assay several aspects of cell death in cultured cells from Atlantic salmon infected with ISAV. Both morphological and biochemical assays have been performed; addressing the effect of ISAV infection on cell viability, DNA fragmentation, plasma membrane alterations and permeability, mitochondrial morphology and caspase activity. To verify the different assays, staurosporine was used as an apoptosis inducing agent. In addition, transcriptional changes in cells infected with different ISAV isolates were investigated.

Both the extrinsic and intrinsic pathways of apoptosis can induce mitochondrial changes. The mitochondria are central to the cells energy metabolism and have been shown to be important in regulation of cell death. Mitochondrial structure is typically arranged as a reticulum. It is shown for several viruses that they interfere with mitochondrial functions; and that mitochondrial redistribution and morphological alterations can occur [45]. Irrespective of mechanism leading to cell death, mitochondrial morphology was altered, as shown by staining with mitotracker (figure 1). This was evident 3 days p.i. with ISAV. The transition from a thread like to a grain like morphology might be due to increased fission or decreased fusion of mitochondria, or possibly a combination or the two. Mitochondrial fragmentation has also been reported as a response to Cytomegalovirus [46], Herpes simplex virus infection [47] and after Rana grylio virus in fish cells [48]. The molecular mechanism and biological significance of this event in ISAV infected cells was not elucidated. We have previously shown that the level of reduced glutathione, GSH, decreases during the course of ISAV infection [31]. We can only speculate whether the observed phenomenon is due to ROS. Staurosporine, which has been shown to act via the mitochondrial pathway [49], was used as an inducer of apoptosis in this model system. SS treatment also induced fragmentation of mitochondria in ASK cells (figure 1, lower panel), in agreement with previous reports using both mammalian and fish cells [50,51].

The mechanisms that regulate apoptosis are complicated. We observed that caspase 3/7 activity increased in all cell types after infection. However, there were differences between cell types with SHK-1 cells showing the highest increase. Although all three cell lines originate from the same tissue (head kidney), our data (caspase activity, metabolic activity) suggests that these cells respond in a different way to ISAV infection, corroborating earlier findings [13]. For influenza virus it has been shown that caspase -3 activation was essential for virus propagation [52]. Joseph et al has previously reported that apoptosis after ISAV infection was caspase 3 dependent [13]. Efforts to discriminate between apoptosis through the extrinsic- (caspase-8 dependent) or intrinsic pathways (caspase-9 dependent) by analysis of these enzymes did not lead to any conclusions, due to high background activity in non-infected cells. Efforts to immunodetect apoptosis-inducing factor (AIF) (immunofluorescent staining) and PARP cleavage (western blotting), were also unsuccessful due to non-specific antibodies. The events leading to caspase 3/7 activation and the downstream effects are yet to be elucidated.

Internucleosomal DNA degradation, resulting in DNA "laddering" when separated on an agarose gel, is a hallmark of apoptosis. In this *in vitro *model, DNA laddering could be induced by SS treatment, but was not apparent in any of the three cell lines infected with ISAV 4. This is in contrast with previous results [13] where laddering was detected in SHK-1 cells infected with a different strain at a higher dose. This difference could therefore be strain- or dose dependent. In a report by Malatova *et al *[53], it was shown that DNA fragmentation in less than 5% of the cells is difficult to detect. To assay DNA fragmentation after ISAV infection by microscopy, TUNEL staining was also performed. The results showed that there were equal numbers of TUNEL positive cells among the mock and ISAV infected (~1%). This might be due to the fact that the TUNEL positive cells detach from the substratum due to CPE and are lost from the assay during washing.

Clearance of damaged or potentially harmful cells, e.g. virus infected, is important to avoid inflammation. During apoptosis, alterations of the plasma membrane phospholipid distribution marks cells for uptake by phagocytosis [20,54]. Using FACS analysis of mock and ISAV infected cells labelled with Annexin V, we compared the effect of viral strains on this parameter in ASK cells. As PS translocation also occurs during necrosis, co-staining with a nucleic acid stain is necessary to determine at what stage of apoptosis the cells are in. The results showed that most cells were viable (80%). The least virulent strain (ISAV 10) caused the lowest mortality. Based on the staining pattern observed by flow cytometry, the cell population positive for AV could be characterized as late apoptotic or necrotic. However, also a proportion of the mock-infected cells were PI positive after staining. This was in contrast to adherent cells that were completely PI negative. When adherent cells were stained with PI and examined by microscopy, both ISAV and mock-infected cells were PI negative. YO-PRO-1, a nucleic acid dye reported to stain apoptotic cells [36], showed progressively more YO-PRO-1 positive cells in the ISAV infected cells over time, thus a clear change in the membrane permeability. Also SS treated cells were YO-PRO-1 positive. Nevertheless, the PI dye was excluded. As apoptotic cells are efficiently phagocytized *in vivo*, no inflammatory response is elicited. *In vitro*, where no phagocytic cells are present, cells ultimately swell and lyse, in a process termed "secondary necrosis", where cells are both AV and PI positive. The fact that uninfected cells also become PI positive in the flow analysis may be due to permeability caused by some reagent in the AV staining kit, as we did not observe PI positive cells in adherent mock-infected cells. Cells that had detached from the substratum due to high cell density in the flasks might also account for the PI positive mock cells.

Previous studies trying to link viral strain virulence to changes in gene expression of immune- and stress-related genes have shown divergent results [55-57]. When mice were infected with different strains of influenza, there was a good correlation between virulence and immune gene up-regulation in lung tissue [55]. Pathway analysis of expression changes revealed that cell death-, interferon- and Toll receptor- pathways were most strongly up-regulated by the most virulent influenza strain. However, other studies comparing wild type and attenuated strains of yellow fever virus [57] and rabies virus [28] have shown that the attenuated strain was a stronger inducer of innate immune genes and responses like apoptosis *in vitro*. The only transcript coming close to being correlated (r = -0,86) with mortality in this investigation was HSP 70. However, genes in common pathways were strongly correlated. In a previous report, we found that genes in the antioxidant-responsive element pathway regulated by Nrf2 were significantly activated by ISAV infection *in vitro *[31]. The main difference between the strains is in the HPR region of the HE protein, except for ISAV 4 and 7 which share the same HPR (but have 33 other variant amino acids) [58]. In a recent report, virulence was mainly correlated with HPR sequence and a Q/H to L substitution in position 266 of the fusion protein (segment 5) [58]. However, there were also amino acid differences in the polymerase subunits, nucleoprotein, matrix protein, both segment 7 OFR's and segment 8 ORF 2 protein of high and low virulent strains.

## Conclusion

Our study has focused on trying to elucidate the mode of cell death in ISAV infected cells. In this model system some assays showed a clear response to apoptosis and stress (caspase 3/7 activity, mitochondrial morphology, uptake of YO-PRO-1 dye, induction of IFN responsive genes), while other assays did not show classic apoptosis responses (DNA laddering, TUNEL, phospholipid distributon). Some responses were cell type specific (caspase 3/7 activity), while other responses were dependent on virus strain (transcriptional responses, phospholipid distributon). It would be interesting to further elucidate the molecular mechanisms of ISAV induced cell death in cells from Atlantic salmon, and also to compare it with the *in vivo *situation. Nevertheless, these results represent a contribution to further define this *in vitro *model system for ISAV infection.

## Methods

### Cell culture and virus

Cell lines from Atlantic salmon (SHK-1, TO and ASK) were maintained in L-15 medium (Leibovitz, Verviers, Belgium), supplemented with 5% (SHK-1 and TO) or 10% (ASK) FCS, 4 mM L-glutamine, 50 μg ml^-1 ^gentamicin sulphate and 40 μM β-mercaptoethanol and were cultured at 20°C. After ISAV infection, the cells were cultured at 15°C. ISAV isolates, named ISAV 2, 4,7 and 10 by Mjaaland et al. [6] were kindly provided by Birgit Dannevig, National Veterinary Institute, Oslo, Norway. When injected into salmon, ISAV 2 and 4 results in high mortality (75 and 67,5% respectively, whereas 7 and 10 display low mortality (7,5 and 6,6% respectively) [6]. For virus production, ASK cells were inoculated with a 1:50 dilution of the isolates at 15°C, and the virus was allowed to adsorb for 4 hours, before cell culture media with 5% serum was added. Supernatants from the cell flasks were collected after 14 days, cleared by centrifugation at 4000 × g for 20 min., aliquoted and frozen at -80°C. For *in vitro *infection of cells, cells were seeded in either 96- or 6-well dishes, or 25 cm^2 ^flasks and grown for 48 hours. Aliquots of the ISAV isolates were thawed in ice water and diluted in serum free L-15 to achieve a MOI (multiplicity of infection) of 1 based on TCID_50 _values obtained by titration as described in [3]. For infection, cells (1.7 × 10^4 ^cells/cm^2^, passages 40–50) were seeded in 25-cm^2 ^flasks and grown in L-15+ medium (Cambrex Bio Sciences, Verviers, Belgium) supplemented with 50 μg ml^-1 ^of gentamicin, 4 mM l-glutamine, 40 μM β-mercaptoethanol, and 10% fetal calf serum for 24 hours at 15°C, then washed four times with serum-free medium and inoculated with ISAV (MOI = 1) for 4 hours (15°C). Cells were harvested at days 1, 3, 5, 7, and 9 post-infection (cytopathogenic effect [CPE] occurred at day 10) and mock-infected controls at days 1, 5, and 9 days post-infection Virus titers in supernatants from ASK cells infected with ISAV 4 on day 5 p.i were determined by end-point titration in ASK cells described in [6].

### Viability assay

Cells were seeded in white 96-well plates in triplicates (1 × 10^3 ^cells per well), allowed to attach and grow for 48 hours at 20°C, and treated with 3 different concentrations of staurosporine (0.5/1/10 μM) for 24 or 48 h or DMSO (vehicle) for 48 h; or infected with ISAV (isolates 2, 4, 7 and 10) or mock for 1, 3, 5, 7 or 9 days. At these time points, cell viability was analyzed using the fluorescence-based Cell Titer Blue (Promega, Madison, WI, USA.) The assay measures the metabolic activity in cells, based on the resazurin to resorufin (fluorescent) reduction reaction. Cell Titer Blue reagent was added to the cells 4 h prior to measurement. Viability was measured at 531_Ex_/615_Em _nm in a Victor^3 ^multilabel plate counter (PerkinElmer, Germany). Mean relative fluorescence ± SE was plotted.

### Caspase activity assay

Caspase 3 and 7 activity was measured in the three cell lines using the Caspase-Glo 3/7 assay kit (Promega, Madison, WI, USA) according to the manufacturer's protocol. Briefly, Caspase-Glo 3/7 reagent was added to the wells directly after viability measurement. The plate was shaken for 30 seconds before measurement of caspase activity every 20 min for 6 h using luminescence settings in the Victor^3 ^plate counter. The slope for each well was calculated and the mean value ± SE was plotted.

### DNA laddering assay

To detect 200 base pair (bp) DNA fragmentation, DNA was isolated from floating and adherent SS (1 μg 24 h), mock or ISAV infected (ISAV 4) SHK-1, TO or ASK cells on days 3, 5 and 7 p.i., using the Apoptotic DNA-Ladder Kit (Roche Diagnostics GmbH, Mannheim, Germany). A positive control for laddering was included in the kit. 2 μg of isolated DNA was electrophoresed on a 1% agarose gel.

### TUNEL staining

To detect endonuclease cleavage of DNA, TUNEL (Terminal deoxynucleotidyl transferase biotin-dUTP nick end labeling) staining was performed in ASK cells using the In Situ Cell Death Detection Kit, Fluorescein (Roche Diagnostics GmbH, Mannheim, Germany). 1.5 × 10^5 ^cells were seeded on glass coverslips in 6-well plates. SS treated (0.5/1/10 μM) for 24 or 48 h, DMSO, mock or ISAV infected (isolate 2, 4, 7 and 10) cells were washed with PBS and fixed in 4% paraformaldehyde on days 3, 5 and 7 p.i. TUNEL staining was performed according to the manufacturer's instructions. The coverslips were mounted using ProLong Gold antifade reagent with DAPI (Invitrogen, molecular Probes, Eugene, OR, USA)

### Flow cytometry

ASK cells (4 × 10^5^) were mock or ISAV infected (isolate 2, 4, 7 and 10) in 25 cm^2 ^flasks for 5 days. To investigate phospatidylserine exposure on the plasma membrane, media containing detached cells was decanted into 15 ml falcon tubes. Adherent cells were washed twice with ice cold PBS and trypsin-EDTA (Invitrogen, Carlsbad, CA, USA) was added. The trypsinized cells and media containing detached cells were pooled and pelleted by centrifugation. The staining with Annexin V-FITC was performed according to the manufacturer's instructions, using the rapid protocol in the Annexin V-FITC Apoptosis Detection Kit (Merck, La Jolla, CA, USA). Propidium iodide (PI) was added to the tubes shortly before examination by flow cytometry. The cells were analyzed on a Becton Dickinson FACS Calibur™ flow cytometer.

### Cell membrane integrity assay

With the same conditions as for the flow cytometry assay, but including days 1, 3, 5, 7 and 9, membrane integrity was assayed in ISAV 4 infected cells by adding the nuclear stains YO-PRO^®^-1 (1 μM), Hoechst 33342 (10 μM) and PI (4,6 μg/ml) to each well after washing the cells 1× with L-15 medium. One hour after addition of the dyes, the cells were examined under a fluorescence microscope.

### Visualization of mitochondria

Mitotracker green FM or Mitotracker Red CMXRos (Invitrogen, molecular Probes, Eugene, OR, USA) at a final concentration of 500 nM and Hoechst 33342 (membrane permeable) at a final concentration of 10 μM (Invitrogen, molecular Probes, Eugene, OR, USA) was mixed in L-15 medium without phenol red and added to ASK cells grown in chambered coverglasses (Nalge Nunc International, Roskilde, Denmark). Cells were treated with 3 different concentrations of staurosporine (0.5/1/10 μM) for 24 or 48 h or DMSO (vehicle) for 48 h; or with ISAV 4 or mock for 1, 3, 5, 7 or 9 days. The cells were washed once in L-15 medium before addition of the dyes. One hour after incubation with the dyes, the cells were examined under a confocal fluorescence microscope, using the 405 nm and 488 nm lasers, and the 543 nm laser for DIC microscopy.

### Real-time quantitative PCR (qPCR)

ASK cells were seeded in 25 cm^2 ^flasks and mock or ISAV infected (isolates 2, 4, 7 and 10) as above. On day 3 p.i., RNA was isolated, using the RNeasy kit (Qiagen, Valencia, CA, USA). The RNA isolation and cDNA synthesis was performed as in [37], except that the reagents was from Roche, and that the samples were run using the Light cycler (Roche). Primers for the analysis of various transcripts are shown in table 1. The reference genes used in this study have previously been validated [59]. Virus induced changes in gene expression was calculated using the REST algorithm whereas differences in gene expression between isolates was analyzed by one way ANOVA. Primer sequences are listed in table S1 in Additional file 1.

**Table 1:** Relative gene expression in ASK cells 3 days p.i. normalized to 2 housekeeping genes (EF1α and 18S)

	**ISAV 2**		**ISAV4**		**ISAV 7**		**ISAV 10**	
**Gene**	**Mean**	**± SD**	**Mean**	**± SD**	**Mean**	**± SD**	**Mean**	**± SD**

***Ifn-α***	27,0^a^	7,6	175,1^b^	54,9	78,8^c^	25,3	53,7^c^	16,3
***Mx-1***	39,0^a^	5,8	105,1^b^	13,3	82,0^b^	14,3	52,5^a^	8,7
***Isg-15***	702,4*	135,0	1720,0*	311,6	1315,5*	303,6	1068,5*	217,5
***p62***	5,7*	0,8	3,2*	0,5	8,5*	1,7	6,7*	1,0
***Galectin-9***	14,2^a^	1,9	21,9^b^	3,1	20,0^b^	3,3	20,8^b^	3,2
***Ciap-1***	2,4^ac^	0,5	2,5^ab^	0,5	3,4^b^	0,8	2,1^c^	0,4
***Caspase-3a***	1,2*	0,1	1,1	0,1	1,2*	0,2	1,1	0,2
***p53***	0,5^a^	0,1	0,7^b^	0,1	0,4^a^	0,1	0,5^a^	0,1
***Hsp-70***	41,8^a^	5,0	26,9^a^	4,3	56,0^b^	11,2	71,2^b^	9,3
***Hsp-90***	1,0	0,1	0,8*	0,1	0,9*	0,1	1,0	0,1
***Transaldolase-1***	0,2^a^	0,0	0,4^b^	0,1	0,2^a^	0,0	0,2^a^	0,0
***Tnf-α***	16,4*	9,6	27,2*	16,3	47,3*	27,5	27,8*	15,8
***Il-1β***	8,3*	5,9	3,7	2,8	11,9*	9,2	9,7*	6,9
***Bcl-2***	1,1	0,2	0,9	0,2	1,2	0,3	1,0	0,2
***Mcl-1***	1,1	0,1	1,4*	0,2	1,6*	0,3	1,4*	0,2
***Ogf-1***	4,5^a^	0,6	6,9^b^	0,9	6,8^b^	1,2	4,8^a^	0,7
***Pdcd-5***	1,1	0,1	0,7*	0,1	1,0	0,2	1,0	0,1
***Fip-2***	1,8*	0,4	2,0*	0,4	2,4*	0,5	1,9*	0,3
***Cox-2***	7,5*	3,3	3,9*	1,9	10,9*	5,3	9,5*	4,3
***Nfκ-β***	1,8*	0,2	2,0*	0,3	3,0*	0,5	2,0*	0,3
***Rig-1***	3,9*	1,4	5,5*	1,7	5,7*	1,9	4,5*	1,4

*Genes that were significantly different from mock are marked with an asterisk.

^a, b, c^Genes that were both different from mock and varied with isolate are marked with letters.

### Statistical analysis

Statistical analysis was performed using the SPSS and Sigma Stat programs. A P value of < 0.05 was considered significant.

## Competing interests

The authors declare that they have no competing interests.

## Authors' contributions

BLS conducted all the experiments except for real-time PCR (LCP) and operation of the FACS machine (ESB). TG, SM and ESB helped in designing the experiments BLS wrote the manuscript. TG and SM edited the manuscript. All authors read and approved the final manuscript.

## Supplementary Material

Additional file 1 List of primers used for real-time qPCR in this study. Click here for file

## References

[B1] Kawaoka Y, Cox NJ, Haller O, Hongo S, Kaverin N, Klenk H-D, Lamb RA, McCauley J, Palese P, Rimstad E, Webster RG, Fauquet CM, Mayo MA, Maniloff J, Desselberger U, Ball LA (2005). Orthomyxoviridae. Virus Taxonomy, Classification and Nomenclature of Viruses, Eight report of the International Committee on the Taxonomy of Viruses.

[B2] Hammell KL, Dohoo IR (2005). Mortality patterns in infectious salmon anaemia virus outbreaks in New Brunswick, Canada. J Fish Dis.

[B3] Lyngstad TM, Jansen PA, Sindre H, Jonassen CM, Hjortaas MJ, Johnsen S, Brun E (2008). Epidemiological investigation of infectious salmon anaemia (ISA) outbreaks in Norway 2003–2005. Prev Vet Med.

[B4] Bouchard D, Keleher W, Opitz HM, Blake S, Edwards KC, Nicholson BL (1999). Isolation of infectious salmon anemia virus (ISAV) from Atlantic salmon in New Brunswick, Canada. Dis Aquat Organ.

[B5] Lovely JE, Dannevig BH, Falk K, Hutchin L, MacKinnon AM, Melville KJ, Rimstad E, Griffiths SG (1999). First identification of infectious salmon anaemia virus in North America with haemorrhagic kidney syndrome. Dis Aquat Organ.

[B6] Mjaaland S, Markussen T, Sindre H, Kjoglum S, Dannevig BH, Larsen S, Grimholt U (2005). Susceptibility and immune responses following experimental infection of MHC compatible Atlantic salmon (Salmo salar L.) with different infectious salmon anaemia virus isolates. Arch Virol.

[B7] Ritchie RJ, McDonald JT, Glebe B, Young-Lai W, Johnsen E, Gagne N (2009). Comparative virulence of Infectious salmon anaemia virus isolates in Atlantic salmon, Salmo salar L. J Fish Dis.

[B8] Jones SRM, MacKinnon AM, Groman DB (1999). Virulence and pathogenicity of infectious salmon anemia virus isolated from farmed salmon in Atlantic Canada. Journal of Aquatic Animal Health.

[B9] Jones SRM, Groman DB (2001). Cohabitation transmission of infectious salmon anemia virus among freshwater-reared Atlantic salmon. Journal of Aquatic Animal Health.

[B10] Raynard RS, Snow M, Bruno DW (2001). Experimental infection models and susceptibility of Atlantic salmon Salmo salar to a Scottish isolate of infectious salmon anaemia virus. Dis Aquat Organ.

[B11] Rolland JB, Winton JR (2003). Relative resistance of Pacific salmon to infectious salmon anaemia virus. J Fish Dis.

[B12] Thorud KE, Djupvik HO (1988). Infectious anemia in Atlantic salmon (*Salmo salar *L.). Bull Eur Assoc Fish Pathol.

[B13] Joseph T, Cepica A, Brown L, Ikede BO, Kibenge FS (2004). Mechanism of cell death during infectious salmon anemia virus infection is cell type-specific. J Gen Virol.

[B14] Everett H, McFadden G (1999). Apoptosis: an innate immune response to virus infection. Trends Microbiol.

[B15] Brydon EW, Morris SJ, Sweet C (2005). Role of apoptosis and cytokines in influenza virus morbidity. FEMS Microbiol Rev.

[B16] Fesq H, Bacher M, Nain M, Gemsa D (1994). Programmed cell death (apoptosis) in human monocytes infected by influenza A virus. Immunobiology.

[B17] Hinshaw VS, Olsen CW, Dybdahl-Sissoko N, Evans D (1994). Apoptosis: a mechanism of cell killing by influenza A and B viruses. J Virol.

[B18] Takizawa T, Matsukawa S, Higuchi Y, Nakamura S, Nakanishi Y, Fukuda R (1993). Induction of programmed cell death (apoptosis) by influenza virus infection in tissue culture cells. J Gen Virol.

[B19] Kerr JF, Wyllie AH, Currie AR (1972). Apoptosis: a basic biological phenomenon with wide-ranging implications in tissue kinetics. Br J Cancer.

[B20] Fadok VA, Voelker DR, Campbell PA, Cohen JJ, Bratton DL, Henson PM (1992). Exposure of phosphatidylserine on the surface of apoptotic lymphocytes triggers specific recognition and removal by macrophages. J Immunol.

[B21] Benedict CA, Norris PS, Ware CF (2002). To kill or be killed: viral evasion of apoptosis. Nat Immunol.

[B22] Hay S, Kannourakis G (2002). A time to kill: viral manipulation of the cell death program. J Gen Virol.

[B23] Inohara N, Nunez G (2000). Genes with homology to mammalian apoptosis regulators identified in zebrafish. Cell Death Differ.

[B24] Takle H, Andersen O (2007). Caspases and apoptosis in fish.

[B25] Takle H, McLeod A, Andersen O (2006). Cloning and characterization of the executioner caspases 3, 6, 7 and Hsp70 in hyperthermic Atlantic salmon (Salmo salar) embryos. Comp Biochem Physiol B Biochem Mol Biol.

[B26] Bereiter-Hahn J, Voth M (1994). Dynamics of mitochondria in living cells: shape changes, dislocations, fusion, and fission of mitochondria. Microsc Res Tech.

[B27] Frank S, Gaume B, Bergmann-Leitner ES, Leitner WW, Robert EG, Catez F, Smith CL, Youle RJ (2001). The role of dynamin-related protein 1, a mediator of mitochondrial fission, in apoptosis. Dev Cell.

[B28] Morimoto K, Hooper DC, Spitsin S, Koprowski H, Dietzschold B (1999). Pathogenicity of different rabies virus variants inversely correlates with apoptosis and rabies virus glycoprotein expression in infected primary neuron cultures. J Virol.

[B29] Lin Y, Bright AC, Rothermel TA, He B (2003). Induction of apoptosis by paramyxovirus simian virus 5 lacking a small hydrophobic gene. J Virol.

[B30] Itoh M, Hotta H, Homma M (1998). Increased induction of apoptosis by a Sendai virus mutant is associated with attenuation of mouse pathogenicity. J Virol.

[B31] Schiotz BL, Jorgensen SM, Rexroad C, Gjoen T, Krasnov A (2008). Transcriptomic analysis of responses to infectious salmon anemia virus infection in macrophage-like cells. Virus Res.

[B32] Bertrand R, Solary E, O'Connor P, Kohn KW, Pommier Y (1994). Induction of a common pathway of apoptosis by staurosporine. Exp Cell Res.

[B33] Weil M, Jacobson MD, Coles HS, Davies TJ, Gardner RL, Raff KD, Raff MC (1996). Constitutive expression of the machinery for programmed cell death. J Cell Biol.

[B34] Cohen GM (1997). Caspases: the executioners of apoptosis. Biochem J.

[B35] Van Cruchten S, Broeck W Van Den (2002). Morphological and biochemical aspects of apoptosis, oncosis and necrosis. Anat Histol Embryol.

[B36] Idziorek T, Estaquier J, De Bels F, Ameisen JC (1995). YOPRO-1 permits cytofluorometric analysis of programmed cell death (apoptosis) without interfering with cell viability. J Immunol Methods.

[B37] Jorgensen SM, Syvertsen BL, Lukacs M, Grimholt U, Gjoen T (2006). Expression of MHC class I pathway genes in response to infectious salmon anaemia virus in Atlantic salmon (Salmo salar L.) cells. Fish Shellfish Immunol.

[B38] Collins CM, Olstad K, Sterud E, Jones CS, Noble LR, Mo TA, Cunningham CO (2007). Isolation of a FIP2-like gene from Atlantic salmon (Salmo salar L.), found upregulated following infection with the monogenean parasite Gyrodactylus salaris Malmberg, 1957. Fish Shellfish Immunol.

[B39] Matejusova I, Felix B, Sorsa-Leslie T, Gilbey J, Noble LR, Jones CS, Cunningham CO (2006). Gene expression profiles of some immune relevant genes from skin of susceptible and responding Atlantic salmon (Salmo salar L.) infected with Gyrodactylus salaris (Monogenea) revealed by suppressive subtractive hybridisation. Int J Parasitol.

[B40] Ingerslev HC, Cunningham C, Wergeland HI (2006). Cloning and expression of TNF-alpha, IL-1beta and COX-2 in an anadromous and landlocked strain of Atlantic salmon (Salmo salar L.) during the smolting period. Fish Shellfish Immunol.

[B41] Best SM (2008). Viral Subversion of Apoptotic Enzymes: Escape from Death Row.

[B42] Imajoh M, Sugiura H, Oshima S (2004). Morphological changes contribute to apoptotic cell death and are affected by caspase-3 and caspase-6 inhibitors during red sea bream iridovirus permissive replication. Virology.

[B43] Hong JR, Wu JL (2002). Induction of apoptotic death in cells via Bad gene expression by infectious pancreatic necrosis virus infection. Cell Death Differ.

[B44] Lin PW, Huang YJ, John JA, Chang YN, Yuan CH, Chen WY, Yeh CH, Shen ST, Lin FP, Tsui WH, Chang CY (2008). Iridovirus Bcl-2 protein inhibits apoptosis in the early stage of viral infection. Apoptosis.

[B45] Chen W, Calvo PA, Malide D, Gibbs J, Schubert U, Bacik I, Basta S, O'Neill R, Schickli J, Palese P (2001). A novel influenza A virus mitochondrial protein that induces cell death. Nat Med.

[B46] McCormick AL, Smith VL, Chow D, Mocarski ES (2003). Disruption of mitochondrial networks by the human cytomegalovirus UL37 gene product viral mitochondrion-localized inhibitor of apoptosis. J Virol.

[B47] Murata T, Goshima F, Daikoku T, Inagaki-Ohara K, Takakuwa H, Kato K, Nishiyama Y (2000). Mitochondrial distribution and function in herpes simplex virus-infected cells. J Gen Virol.

[B48] Huang YH, Huang XH, Gui JF, Zhang QY (2007). Mitochondrion-mediated apoptosis induced by Rana grylio virus infection in fish cells. Apoptosis.

[B49] Kruman I, Guo Q, Mattson MP (1998). Calcium and reactive oxygen species mediate staurosporine-induced mitochondrial dysfunction and apoptosis in PC12 cells. J Neurosci Res.

[B50] Cribbs JT, Strack S (2007). Reversible phosphorylation of Drp1 by cyclic AMP-dependent protein kinase and calcineurin regulates mitochondrial fission and cell death. EMBO Rep.

[B51] Kim MJ, Kang KH, Kim CH, Choi SY (2008). Real-time imaging of mitochondria in transgenic zebrafish expressing mitochondrially targeted GFP. Biotechniques.

[B52] Wurzer WJ, Planz O, Ehrhardt C, Giner M, Silberzahn T, Pleschka S, Ludwig S (2003). Caspase 3 activation is essential for efficient influenza virus propagation. Embo J.

[B53] Matalova ESA (2002). Detection of Apoptotic DNA Ladder in Pig Leukocytes and its Precision Using LM-PCR (Ligation Mediated Polymerase Chain Reaction). ACTA VETERINARIA BRNO.

[B54] Martin SJ, Reutelingsperger CP, McGahon AJ, Rader JA, van Schie RC, LaFace DM, Green DR (1995). Early redistribution of plasma membrane phosphatidylserine is a general feature of apoptosis regardless of the initiating stimulus: inhibition by overexpression of Bcl-2 and Abl. J Exp Med.

[B55] Kash JC, Tumpey TM, Proll SC, Carter V, Perwitasari O, Thomas MJ, Basler CF, Palese P, Taubenberger JK, Garcia-Sastre A (2006). Genomic analysis of increased host immune and cell death responses induced by 1918 influenza virus. Nature.

[B56] Ray N, Enquist LW (2004). Transcriptional response of a common permissive cell type to infection by two diverse alphaherpesviruses. J Virol.

[B57] Lefeuvre A, Contamin H, Decelle T, Fournier C, Lang J, Deubel V, Marianneau P (2006). Host-cell interaction of attenuated and wild-type strains of yellow fever virus can be differentiated at early stages of hepatocyte infection. Microbes Infect.

[B58] Markussen T, Jonassen CM, Numanovic S, Braaen S, Hjortaas M, Nilsen H, Mjaaland S (2008). Evolutionary mechanisms involved in the virulence of infectious salmon anaemia virus (ISAV), a piscine orthomyxovirus. Virology.

[B59] Jorgensen SM, Kleveland EJ, Grimholt U, Gjoen T (2006). Validation of reference genes for real-time polymerase chain reaction studies in Atlantic salmon. Mar Biotechnol (NY).

