# Supplementary material for: Analysis of host- and strain-dependent cell death responses during infectious salmon anemia virus infection in vitro
Source: Virol J. 2009 Jul 1;6:91. doi: 10.1186/1743-422X-6-91 (PMC2715388; doi:10.1186/1743-422X-6-91)
Supplement: Additional file 1 — List of primers used for real-time qPCR in this study. [file 1743-422X-6-91-S1.doc]

| **Gene** | **Dir.** | **Sequence** | **E**^†^ | **Amplicon** | **Acc.nr.** |
| --- | --- | --- | --- | --- | --- |
| ***18S*** | F | TGTCCGCTAGAGGTGAAATT | 2,00 | 61 | AJ427629 |
|  | R | GCAAATGCTTTCGCTTTCG |  |  |  |
| ***Ef1-α*** | F | CACCACCGGCCATCTGATCTACAA | 1,97 | 77 | AF321836 |
|  | R | TCAGCAGCCTCCTTCTCGAACTTC |  |  |  |
| ***Ifn-α*** | F | CCTGCCATGAAACCTGAGAAGA | 2,00 | 107 | AY216594 |
|  | R | TTTCCTGATGAGCTCCCATGC |  |  |  |
| ***Isg-15*** | F | TGTTAGGTGTCAATGGGAGCAA | 2,03 | 151 | AY926456 |
|  | R | TGTGTCTGGCCCTTTTCGTT |  |  |  |
| ***Mx-1*** | F | TGATCGATAAAGTGACTGCATTCA | 2,00 | 80 | SSU66477 |
|  | R | TGAGACGAACTCCGCTTTTCA |  |  |  |
| ***Tnf-α*** | F | CGGGTTCAAGCTACAAGGGA | 2,21 | 51 | DQ787158 |
|  | R | AAGAGCCCAGTGTGTGGGAT |  |  |  |
| ***Il-1β*** | F | GGAGAGGTTAAAGGGTGGCG | 2,06 | 51 | AY617117 |
|  | R | TCCTTGAACTCGGTTCCCAT |  |  |  |
| ***Ogf-1*** | F | GCAAAGCAGAGGCTGGTCAC | 2,02 | 51 | AM259613 |
|  | R | GCCGTAGAAGTCCAGCATGAG |  |  |  |
| ***Hsp-70*** | F | AGACAGGCCACTAAGGACGC | 1,98 | 51 | AJ632154 |
|  | R | TCTCAGCACATTCAGCCCAG |  |  |  |
| ***Hsp-90*** | F | GAATGACAAGGCGGTGAAGG | 1,97 | 51 | AF135117 |
|  | R | AGAGCGGTCTCGAAAAGCAG |  |  |  |
| ***Cox-2*** | F | GCGGTTTATTCCGGATCCTC | 2,20 | 51 | AY848944 |
|  | R | GCGAAGAAGGCGAACATGAG |  |  |  |
| ***Pdcd-5*** | F | AACCGACGAAAGGTGATGATGGA | 1,98 | 101 | TC29121 |
|  | R | CCTGAATCCCTCCCTAAGCC |  |  |  |
| ***Caspase-3*** | F | GGAATGAGCTTTCGCAATGG | 1,91 | 101 | DQ008069 |
|  | R | ACAGTCTGGTCATTGGCAACC |  |  |  |
| ***Ciap-1*** | F | AGAACAGCCCGTTCCTCATG | 1,98 | 101 | TC43301 |
|  | R | CCGAGCGAAGGTGGAGATAC |  |  |  |
| ***Bcl-2*** | F | TTGTCAGTTGGTGCTGGAGG | 1,93 | 101 | TC29759 |
|  | R | CCAAATTGCTTCTGCCGTTC |  |  |  |
| ***Mcl-1*** | F | TTGACTTGGATGACCGATGC | 2,12 | 51 | AM259614 |
|  | R | TGGCCACAGAATTGATGACG |  |  |  |
| ***Nfκ-β*** | F | ATGGCAGAAGAGGAGCCTTACCTACCT | 1,90 | 81 | TC55326 |
|  | R | AGAGAAATTCAGAGGATCCCATAGTGGA |  |  |  |
| ***Trans-aldolase-1*** | F | ACCTGCTTACCATTTCCCCC | 2,01 | 100 | TC25489 |
|  | R | CAAATCACAGGCCTTGGCTT |  |  |  |
| ***p53*** | F | AAAACCCCCGGCAATAACAA | 2,00 | 151 | BG934348 |
|  | R | CTAGGACCTGAAAGCAGCAGC |  |  |  |
| ***p62*** | F | TGATGCCTCATCTGCTGCC | 2,04 | 102 | CA375694 |
|  | R | CGATACCCAGTGGGCTAAGC |  |  |  |
| ***Fip-2*** | F | CAGCTGCTCCAGTCCCTGAG | 1,99 | 51 | DQ496225 |
|  | R | TTGTAGCCTCTCCACCCTCTG |  |  |  |
| ***Galectin-9*** | F | TTAACCTGCGTTTCAACTCGG | 2,16 | 101 | AF483533 |
|  | R | TGGACCCCACTGTTCCTTCA |  |  |  |

† PCR efficiency
